# Identifying and avoiding radiation damage in macromolecular crystallography

**DOI:** 10.1107/S2059798324003243

**Published:** 2024-04-30

**Authors:** Kathryn L. Shelley, Elspeth F. Garman

**Affiliations:** a Department of Biochemistry, University of Oxford, Dorothy Crowfoot Hodgkin Building, South Parks Road, Oxford OX1 3QU, United Kingdom; b Department of Biochemistry, University of Washington, Seattle, Washington, USA; c Institute for Protein Design, University of Washington, Seattle, Washington, USA; Diamond Light Source, United Kingdom

**Keywords:** radiation damage, specific damage, global damage, *B*_net_, *B*_net_-percentile

## Abstract

Radiation damage remains one of the major impediments to accurate structure solution in macromolecular crystallography. The artefacts of radiation damage can manifest as structural changes that result in incorrect biological interpretations being drawn from a model, they can reduce the resolution to which data can be collected and they can even prevent structure solution entirely. In this article, we discuss how to identify and mitigate against the effects of radiation damage at each stage in the macromolecular crystal structure-solution pipeline.

## Abstract 2

This review covers the symptoms of radiation damage in macromolecular crystallography, how to avoid accruing radiation damage during data collection and how to identify and correct for radiation-damage artefacts in a solved structure.

## 1. Introduction

**Figure 1:**
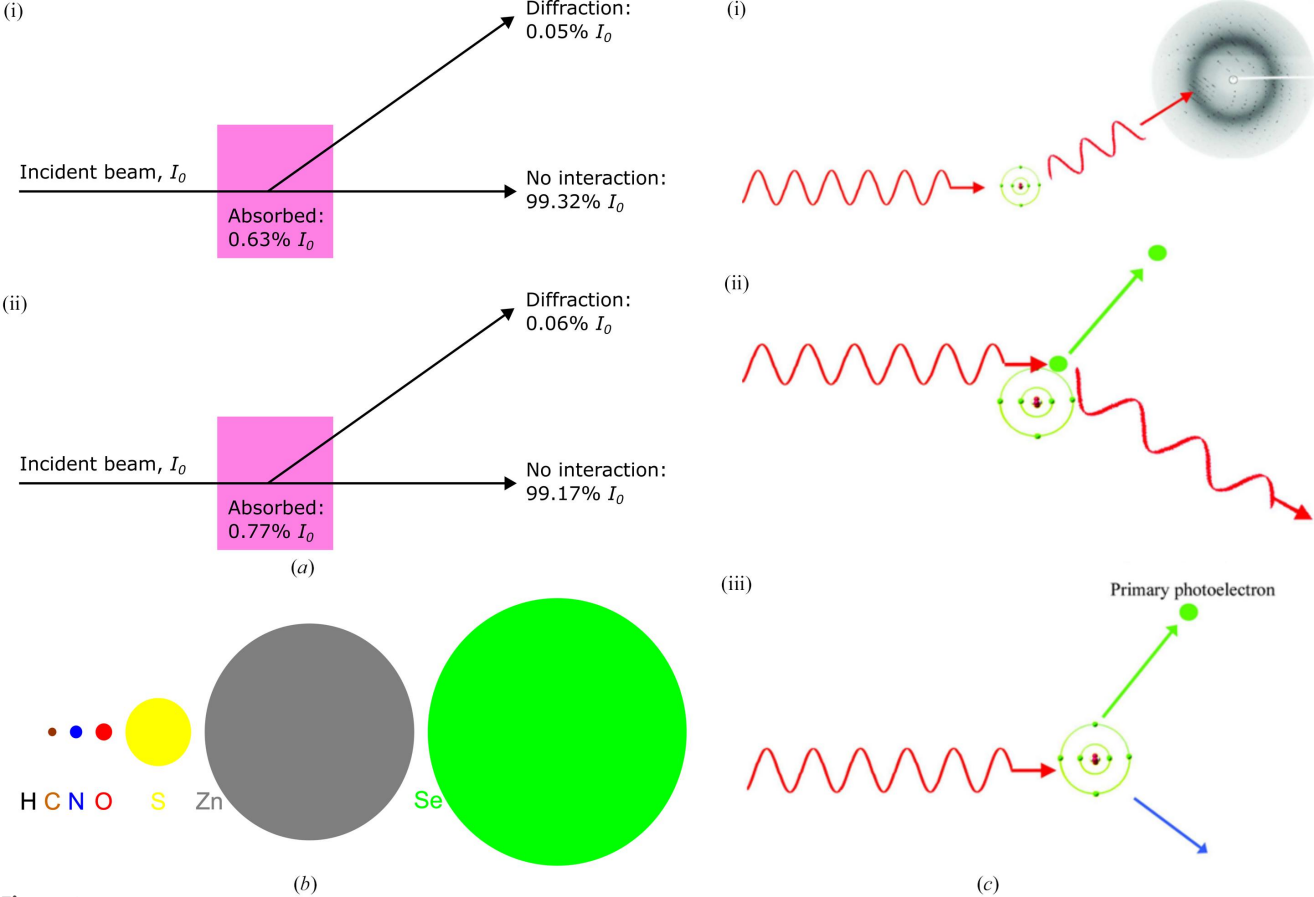
(*a*) Percentages of a 13.0 keV incident beam that interact with a 30 µm thick crystal of metallo-β-lactamase (PDB entry 1znb; Concha *et al.*, 1996 ▸) (i) without and (ii) with Zn. Also shown are the percentages that diffract and are absorbed. (*b*) X-ray photoelectric scattering cross sections for H, C, N, O, S, Zn and Se for 13.0 keV incident X-ray energy. The areas of the circles are proportional to the cross sections [1.85 × 10^−3^ (data not shown), 17.7, 35.7, 64.6, 1217, 12 790 and 19 300 barns per atom, respectively; 1 barn = 10^−28^ m^2^; after Ravelli *et al.*, 2005 ▸]. (*c*) Primary X-ray interaction processes with the atoms of the crystal and solvent. (i) Elastic (aka Thomson, coherent) scattering. The waves add vectorially to give the diffraction pattern. (ii) Compton (aka incoherent) scattering. The X-ray transfers some energy to an atomic electron and thus has lower energy (higher wavelength) after the interaction. (iii) Photoelectric absorption. The X-ray transfers all its energy to an atomic electron, which is then ejected. The excited atom can then emit a characteristic X-ray or an Auger electron to return to its ground state. Reproduced from Garman (2010 ▸).

Diffraction patterns are produced by elastic scattering of incident radiation by a diffraction grating such as a crystal lattice. However, when we irradiate a macromolecular crystal with X-rays, only a tiny minority of the incident X-rays are elastically scattered (Fig. 1 ▸ *a*). In fact, the vast majority pass straight through the crystal. As an example, 99.3% of the X-rays in a 13.0 keV (0.95 Å) beam will pass straight through a 30 µm thick crystal of metallo-β-lactamase (PDB entry 1znb; Concha *et al.*, 1996 ▸) that is missing Zn^2+^ at its two metal-binding sites (and hence does not contain any elements heavier than sulfur). Of the 0.68% of X-rays that interact with the crystal, just 7.9% (0.05% of the total incident beam) are elastically scattered to generate the measured diffraction pattern. The remaining 92.1% (0.63% of the incident beam) of X-rays are inelastically scattered by the crystal, meaning that they deposit some or all of their energy within the crystal, causing damage to it.

Notably, because the X-ray scattering cross section increases in approximate proportion to the fourth power of the atomic number (Garman & Owen, 2006 ▸; Fig. 1 ▸ *b*), crystals containing heavier elements interact with a larger fraction of the incident beam. The same metallo-β-lactamase crystal described earlier but bound to the expected two Zn^2+^ ions per monomer would interact with 0.83% of the incident X-rays, of which 93.3% (0.77% of the incident beam) are inelastically scattered and 6.7% (0.06% of the incident beam) are elastically scattered (Fig. 1 ▸ *a*). Consequently, the introduction of just two Zn^2+^ ions per monomer increases the fraction of X-rays that are inelastically scattered from 0.63% to 0.77%: an increase of approximately 20%. Crystals containing heavier elements are accordingly more susceptible to radiation damage (RD).

Inelastically scattered X-rays deposit energy within a crystal via two mechanisms: the Compton effect and the photoelectric effect (Fig. 1 ▸ *c*). In the Compton effect (Fig. 1 ▸ *c*, ii) the incident X-ray transfers a percentage of its energy to an atomic electron in the crystal, resulting in an excited electron and a lower energy X-ray. The affected electron will be ejected from its parent atom if the energy transferred to the electron exceeds its binding energy to that atom. In the photoelectric effect (Fig. 1 ▸ *c*, iii), the incident X-ray is completely absorbed by an atom, which causes the atom to emit an electron (known as a photoelectron), with the highest probability being for the ejection of this ‘photoelectron’ to be from an atomic inner shell (*K* shell). To return to a lower energy state, an outer shell electron drops down to complete the inner shell; the energy released from the transition is either emitted as a fluorescent X-ray or converted into the release of an outer shell electron (known as an Auger electron; Nave, 1995 ▸).

The electrons released by these inelastic collisions deposit the energy that they carry in the crystal by exciting and/or ionizing atoms in their path, thus damaging the crystal. Each individual electron can affect a large number of atoms: a 13 keV photoelectron, for example, carries sufficient energy to ionize approximately 520 atoms (assuming 25 eV deposited per ionization event; O’Neill *et al.*, 2002 ▸). Moreover, these excitation and ionization events can produce free-radical species that propagate chain reactions of free-radical formation and destruction; in macromolecular crystals these radical species are predominantly hydroxyl radicals as a result of the high solvent content. In addition, the X-rays emitted by both mechanisms can be further inelastically scattered by the crystal to produce more excited/ejected electrons and hence further damage.

The inelastic scattering and absorption of an X-ray beam by a macromolecular crystal is inevitable and is a fact of physics. The fraction of the incident beam that is inelastically scattered can in some cases be reduced, for example by decreasing the heavy-metal content of the crystal if possible, but it cannot be completely prevented. The damage caused to the crystal also cannot be entirely avoided, although there are several steps that a crystallographer can take to greatly reduce the extent to which RD affects their data. RD can be divided into two classes: global damage, which affects the crystal lattice and hence manifests in reciprocal space, and specific damage, which affects the individual asymmetric unit copies and thus occurs in real space. In the following article, we discuss how to identify and mitigate against both of these classes of RD artefacts during the macromolecular crystallography (MX) structure-solution process.

## 2. Global radiation damage

The various manifestations of damage in reciprocal space have been well characterized over the last 20 years and have been reported in the literature for both cryo-temperature and room-temperature (RT) MX diffraction data (for a recent review, see Garman & Weik, 2023 ▸). Arguably the most useful metric against which to quantify the damage rate is the dose, which is defined as the energy absorbed per unit mass of the crystal (J kg^−1^ = gray, Gy). This absorbed dose cannot be directly measured; it can only be estimated from the experimental parameters. This requires knowledge of the characteristics of both the beam (energy, area, profile and flux in photons per second) and also the crystal (size, number of amino acids, heavy-atom content, percentage of solvent and solvent constituents, which allows the crystal absorption coefficients to be computed). Using dose as the *x* axis against which to plot reciprocal-space data analytics allows a much more effective comparison between different beamline conditions and a range of samples, enabling RD effects to be better characterized.

**Figure 2:**
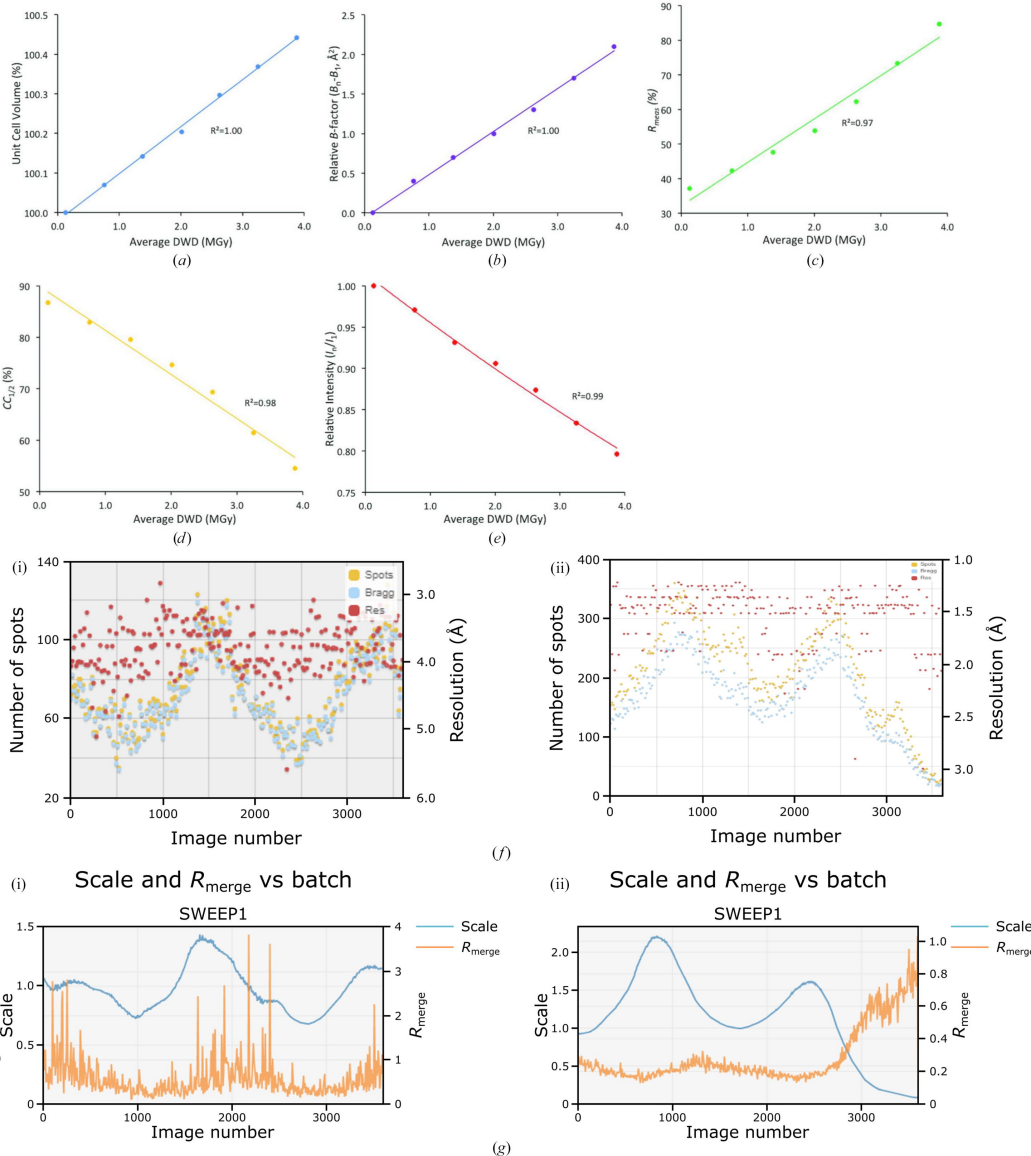
(*a*)–(*e*) Global damage-effect metrics measured for seven successive complete data sets collected from a xylose isomerase crystal at 100 K. (*a*) The unit-cell volume, (*b*) the relative Wilson *B*-factor, (*c*) *R*_meas_, (*d*) CC_1/2_ and (*e*) the relative diffraction intensity as the summed reflection intensity of a data set divided by that of the first data set, *I*_*n*_/*I*_1_, all as a function of average diffraction-weighted dose (Zeldin, Brockhauser *et al.*, 2013 ▸). Reproduced from Taberman *et al.* (2019 ▸). (*f*) *SynchWeb* (Fisher *et al.*, 2015 ▸) display as shown in ISPyB of the automatic analysis of the number of spots and the resolution as a function of image number (rotation angle), which vary as the crystal volume changes during the rotation for data collection from two different protein crystals: (i) an undamaged data set and (ii) a damaged data set demonstrating the characteristic decrease in number of spots by the end of the sweep. (*g*) Scaling factor and *R*_merge_ as a function of image number: (i) an undamaged data set, characterized by the metrics returning to their initial level after 360° rotation (image number 3600), and (ii) a damaged data set, in which *R*_merge_ rises steeply by the end of the collection sweep. A low scaling factor indicates weak diffraction.

As the absorbed dose increases, global damage effects become increasingly evident. These include fading and loss of reflection intensity, with the highest resolution reflections disappearing first, increased Wilson *B*-factor, unit-cell volume expansion, often increased mosaicity, a characteristic ‘U’ or ‘W’ shape (for 180° and 360° wedges, respectively) in the scale applied to each image as a function of rotation angle and decreasing CC_1/2_, plus worsening quality indicators such as *R*_meas_ and *I*/σ(*I*). Some of these pathologies are illustrated in Fig. 2 ▸.

RD rates show some variation between crystals, but for cryocooled crystals some effects usually become visible in the diffraction pattern around dose values of tens of MGy. An experimental dose limit of 30 MGy for 2.4 Å resolution data from cryocooled holo and apoferritin crystal data has been determined as the dose at which the summed reflection intensity fell to 0.7 of its initial value (dose to 0.7, *D*_0.7_). Beyond this dose it was deemed that the biological interpretation of the resulting electron density (see below) was likely to become compromised (Owen *et al.*, 2006 ▸), since at a *D*_0.5_ of 43 MGy many of the amino acids showed specific damage. For lysozyme (HEWL) crystals diffracting to 1.7 Å resolution, a *D*_0.5_ of around 10 MGy has been reported (Teng & Moffat, 2000 ▸), agreeing within error with *D*_0.5_ values of 9 and 12.5 MGy for data to 1.8 Å (De la Mora *et al.*, 2011 ▸) and 1.4 Å resolution (Bugris *et al.*, 2019 ▸), respectively. At RT the radiation decay rate is usually approximately 70 times higher for a similar dose (Nave & Garman, 2005 ▸), and for lysozyme *D*_0.5_ has been determined to be 0.57 MGy for data to 2.0 Å resolution (De la Mora *et al.*, 2020 ▸).

As the above values demonstrate, *D*_0.5_ is a resolution-dependent metric, which results from the fact that higher resolution data fade much faster (lower *D*_0.5_) than lower resolution shells (higher *D*_0.5_; Owen *et al.*, 2006 ▸; Atakisi *et al.*, 2019 ▸). In a survey of all available X-ray and electron dose-limit data at the time, Howells and coworkers suggested a general formula for the relationship between the approximate tolerable dose and resolution: dose (MGy) = 10 × resolution (Å) (for example, for 2.0 Å resolution data this gives 20 MGy; Howells *et al.*, 2009 ▸). Notably, all of these doses are enormous compared with those tolerated by living organisms: a whole-body dose of only 2–10 Gy will cause a rodent to die of internal organ failure in 10–30 days (Coggle, 1983 ▸), whilst the maximum radiotherapy treatment for a human glioblastoma multiforme brain tumour is 60 Gy, divided into 2 Gy doses across 30 days.

It is important to note that these dose limits are just that: limits beyond which further data collection is ill-advised. The protein crystal might well be significantly damaged before the limit is reached, since consideration of dose only accounts for the physics of the interaction of the X-ray beam with the crystal and does not take into account any radiation chemistry effects which may hasten the demise of the crystal order.

### 2.1. How to spot it

The most obvious sign of radiation decay during the experiment is the gradual fading of diffraction intensity on the raw images as they appear. However, if using a modern photon-counting detector (EIGER and PILATUS) and fine-φ (oscillation angle) slicing, for example φ < 0.2°, this can be challenging. These detectors differ substantially from the integrating charge-coupled device (CCD) detectors in that they convert X-rays directly into electronic charge, which is immediately processed electronically with no added noise (*i.e.* there is no ‘readout noise’). Individual photons are counted as they arrive instead of the charge being integrated for a certain time and then read out, and when there are no X-rays they register no background counts (*i.e.* they have no so-called ‘dark current’). When using these detectors, by the time that reflection-intensity fading becomes obvious to the experimenter, it is already too late to avoid deleterious effects on the data. This is due to a number of factors, including the sheer speed of data collection, which means it is not feasible to inspect and compare more than a few diffraction images. In addition, it is hard to judge the true resolution of the data by human eye due to the partial nature of individual reflection intensities per fine-sliced image, since the reflection intensity is split across multiple images. Crystals usually diffract better and can be processed to higher resolution than reflections can be seen on the images.

A better strategy is to monitor the automatic data-processing output as it appears, as well as keeping an eye on the display of the individual images. Signs of radiation decay can be identified during data collection from the per-image plots of the number of Bragg spots and the data resolution, and also from the scale and merging statistic graphs. Fig. 2 ▸(*f*) shows examples of ‘good’ and ‘RD compromised’ output from *DIALS* (Winter *et al.*, 2022 ▸) as run within *xia*2. As the volume of the sample exposed to the beam changes during the goniometer rotation, the number of Bragg peaks oscillates and then returns (Fig. 2 ▸ *f*, i) or does not return (Fig. 2 ▸ *f*, ii) to the maximum as the sample becomes damaged. The scaling outputs for undamaged (Fig. 2 ▸ *g*, i) and damaged (Fig. 2 ▸ *g*, ii) crystals show similar oscillatory behaviour as the sample is rotated. The characteristic ‘W’ shape mentioned above is clearly visible in Fig. 2 ▸(*g*)(ii) as the software attempts to optimize the scales for the majority of the 360° data sweep. Note the correlation between the scales and the number of reflections. *R*_merge_ is also displayed and its sharp increase near the end of the sweep is an obvious sign of significant RD having been suffered by the crystal.

### 2.2. What to do about it

What can an experimenter do to minimize the effects of RD in reciprocal space?

The obvious first step is to try cryocooling the crystal in liquid nitrogen at 77 K, if necessary soaking it first in cryoprotectant (Garman & Schneider, 1997 ▸; Pflugrath, 2015 ▸). Diffraction data are then collected with the crystal bathed in an open cold nitrogen stream. The temperature of the nitrogen should be held below 110 K to prevent the movement of most radical species produced by the absorption of X-rays, including hydroxyl radicals from the radiolysis of water. Above 110 K these latter radicals are thought to become mobile (Owen *et al.*, 2012 ▸) and are the major cause of most RT damage since they are highly reactive and, if mobile, can initiate radiation chemistry reactions in the solvent channels (Southworth-Davies *et al.*, 2007 ▸).

Cryocooling to prolong crystal lifetime has many advantages over RT data collection, including gentler mounting methods, lower background scattering and higher resolution data (since higher resolution reflections do not fade so fast); fewer crystals are required and crystals can easily be shipped in transport Dewars to synchrotrons and can be cryocooled when in peak condition (for example before crystal degradation or before a ‘skin’ appears over the crystal drop which can indicate the formation of oxidized or denatured protein at the air–drop interface) for later data collection. However, it is clear that structures determined at 100 K can lack some relevant structural conformations that only occur at RT (Fraser *et al.*, 2011 ▸), so if these are important to answer the biological question at hand then RT data collection should be carried out. For a recent review discussing the practical aspects of RT data collection and giving information on data-analysis strategies, see Fischer (2021 ▸).

If cryocooled crystals show RD and crystal supply is not an issue, data can be collected from multiple crystals (at cryo-temperature or RT) to reduce the dose absorbed by individual crystals. Excellent software allowing small sections of data or even individual images to be efficiently combined together is now available (for example *xia*2.*multiplex* in *DIALS*; Gildea *et al.*, 2022 ▸) and makes multiple crystal data collection to obtain one complete data set a realistic option. An extreme application of this is expedited at X-ray free-electron laser (XFEL) sources, where a series of X-ray pulses each lasting a few tens of femtoseconds hits a moving stream of RT crystals supplied to the beam (Barends *et al.*, 2022 ▸) and ‘diffraction before destruction’ (Chapman *et al.*, 2014 ▸) usually allows RD-free data to be collected if the pulse is short enough (Nass *et al.*, 2020 ▸). In fact, the so-called ‘serial data collection’ method is increasingly being employed for both structure solution and time-resolved studies (Pearson & Mehrabi, 2020 ▸) and is also employed with sample holders consisting of chips holding thousands of crystals which are moved across the beam (see, for example, Owen *et al.*, 2017 ▸). This modality is likely to grow in popularity with the advent of more fourth-generation sources providing even higher X-ray flux densities.

A very important method to avoid RD is to adopt a data-collection strategy that takes into account the dose absorbed by the samples using the characteristics of the beamline. Several utilities are now available to estimate the dose absorbed during a data collection so that a collection strategy that keeps the dose as low as possible for the whole data set can be adopted. These tools include *DOZOR* at the Massively Automated Sample Selection Integrated Facility (MASSIF-1) beamline of the European Synchrotron Radiation Facility (Svensson *et al.*, 2015 ▸), *RADDOSE*-3*D* (Zeldin, Gerstel *et al.*, 2013 ▸; Bury *et al.*, 2018 ▸) and James Holton’s ‘expected crystal lifetime calculator’ (https://bl831.als.lbl.gov/xtallife.html). In fact, *RADDOSE*-3*D* is now available on several beamlines (for example I04 at Diamond Light Source and all of the MX beamlines at the Stanford Synchrotron Radiation Lightsource) so that exposures can be collected for a specified absorbed dose value rather than for a specified time.

Some rules of thumb born out of experience suggest that if phases are not required, a reasonable data-collection strategy would be to spread a maximum of 10–15 MGy dose over 360–720° in rotation angle, but to not exceed 7 MGy if the crystal diffracts to 1.4 Å resolution or better. If anomalous data are being collected to obtain phase information, however, doses should be kept much lower. Above all, the temptation to increase the exposure time or to reduce the beam attenuation and hence increase the dose should be resisted.

If the crystallization buffer contains any heavier elements such as arsenic (which is present in cacodylate), it can be well worth back-soaking the crystals in a buffer containing lighter elements in which the crystals are stable before cryocooling them or irradiating them at RT. This reduces the absorption coefficient of the crystal and thus lowers the dose for the same irradiation regime, giving more time for data collection before the dose limit is reached and the effects of damage become evident.

Another way to reduce the rate of radiation damage is to use higher incident X-ray energies, as suggested by Nave & Hill (2005 ▸). As the X-ray energy increases, photoelectric cross sections decrease. In addition, there is a higher probability that photoelectrons can escape, especially from microcrystals, and thus not contribute to the absorbed dose. With the recent availability of new CdTe pixel detectors that are able to detect higher energy X-rays with good efficiency, this idea has been experimentally validated (Storm *et al.*, 2020 ▸, 2021 ▸). The observed improvement in diffraction efficiency (the total number of elastically scattered photons/absorbed dose in MGy) was in line with earlier Monte Carlo simulation predictions (Dickerson & Garman, 2019 ▸), and the resolution to which data could be collected for the same dose was better.

If the data-processing statistics show a loss of high-resolution reflections, it is well worth reprocessing the images after cutting out the damaged ones, provided that the data completeness will not suffer too much by doing so. Removing compromised data can lead to substantial improvements in the final electron-density map (see Section 4).

## 3. Specific radiation damage

### 3.1. What is specific damage?

**Figure 3:**
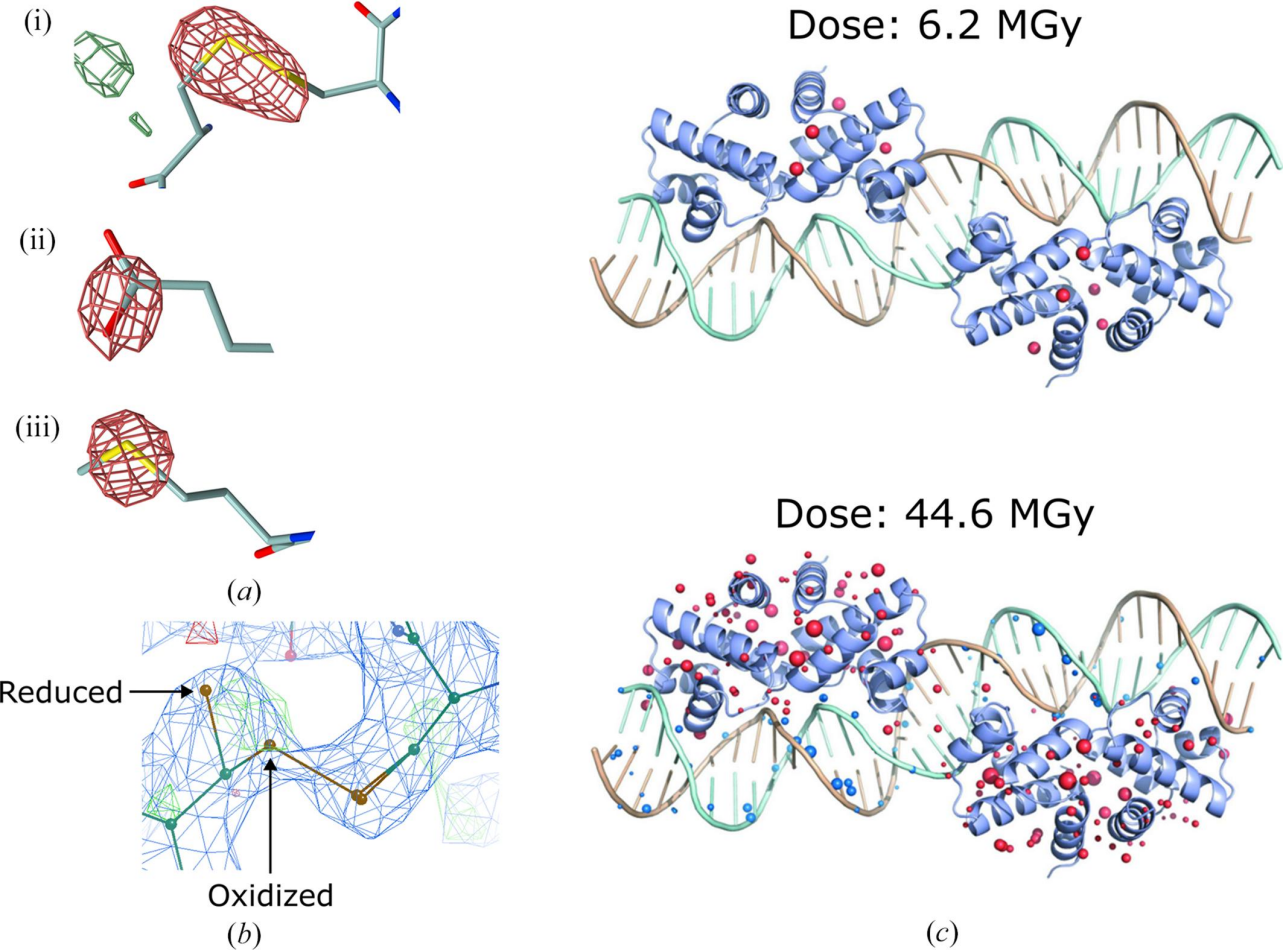
(*a*) Examples of (i) disulfide-bond cleavage, (ii) glutamate decarboxylation and (iii) methionine methylthio group disordering in an *F*_obs(late)_ − *F*_obs(early)_ difference map calculated for a structure of *Torpedo californica* acetylcholinesterase (PDB entry 1qid; Weik *et al.*, 2000 ▸). The maps are contoured at ±4σ, with positive and negative difference density coloured green and red, respectively. This figure was adapted from Bury, Carmichael *et al.* (2016 ▸). (*b*) A disulfide bond modelled in both oxidized and reduced conformations to account for radiation damage in a structure of the tumour necrosis factor protein BAFF bound to a bhpBR3 peptide (PDB entry 3v56; Smart *et al.*, 2012 ▸). The oxidized and reduced conformers of the cysteine side chain that undergoes a large conformational change are indicated with arrows. The 2*mF*_obs_ − *DF*_calc_ map (blue) is contoured at 1.5 r.m.s.d.; the *F*_obs_ − *F*_calc_ difference density map is contoured at ± 3.0 r.m.s.d., with positive and negative density coloured green and red, respectively. (*c*) A representation of the specific damage suffered by low-dose and high-dose data sets collected for the C.Esp1396 protein in complex with its target DNA sequence. Damage artefacts are represented as spheres: the spheres are coloured blue and red depending on whether they are within 2 Å of or further than 2 Å from the DNA, respectively. The radius of each sphere is proportional to the electron-density loss (electrons per Å^3^). This figure was adapted from Bury *et al.* (2015 ▸). All data leading to these structures were collected at 100 K.

Specific damage affects the individual copies of the asymmetric unit, inducing both chemical and structural changes. At cryo-temperatures, specific damage artefacts in protein crystal structures have been observed to occur in a reproducible order with increasing dose (Ravelli & McSweeney, 2000 ▸; Burmeister, 2000 ▸; Weik *et al.*, 2000 ▸). Firstly, metal ions and cofactors are reduced, at doses as low as tens or hundreds of kGy (Beitlich *et al.*, 2007 ▸; Corbett *et al.*, 2007 ▸; Horrell *et al.*, 2016 ▸; Ueno *et al.*, 2019 ▸), before disulfide bonds start to be reduced at doses of approximately 0.5 MGy (Sutton *et al.*, 2013 ▸; De la Mora *et al.*, 2020 ▸). Aspartate and glutamate side chains are then decarboxylated, typically at around doses of 3–4 MGy (Fioravanti *et al.*, 2007 ▸; Bury *et al.*, 2018 ▸). Additional specific RD artefacts include cleavage of the methylthio/methylseleno group from methionine/selenomethionine side chains (Holton, 2007 ▸; Bury *et al.*, 2015 ▸), plus conformational disordering of side chains such as tyrosine, lysine and histidine (Yabukarski *et al.*, 2022 ▸). Moreover, local environment factors such as solvent accessibility, conformational strain, location at an active site and location at a crystal contact have all been found to influence the susceptibility of a residue to these various specific damage artefacts (Dubnovitsky *et al.*, 2005 ▸; Fioravanti *et al.*, 2007 ▸; Holton, 2009 ▸; Gerstel *et al.*, 2015 ▸; Bhattacharyya *et al.*, 2020 ▸). Fig. 3 ▸(*a*) shows examples of some of these damage artefacts in electron-density maps, whilst Fig. 3 ▸(*b*) shows an example case in which a disulfide bond has been modelled in both oxidized and reduced conformations to account for RD.

At cryo-temperatures, the onset of specific damage has been observed at much lower doses than that of global damage (Teng & Moffat, 2000 ▸; Owen *et al.*, 2006 ▸; Gotthard *et al.*, 2019 ▸; De la Mora *et al.*, 2020 ▸). It is therefore possible to collect a data set that contains none of the pathologies described in the preceding section on global damage, yet still contains specific damage artefacts. Conversely, at RT the onset of specific and global damage seems to be less decoupled, although the precise order of onset remains under debate (Roedig *et al.*, 2016 ▸; Gotthard *et al.*, 2019 ▸; De la Mora *et al.*, 2020 ▸). The reason for this difference is that in cryocooled crystals, only the free electrons (plus the propagating holes they leave behind) resulting from inelastic scattering, and also perhaps hydrogen atoms, are able to move (primarily by quantum-mechanical tunnelling) and thus damage the crystal. In contrast, at RT the free-radical species, which are predominantly hydroxyl radicals generated from the radiolysis of water, and solvated electrons produced are able to move as well (Jones *et al.*, 1987 ▸; Symons, 1995 ▸; Zakurdaeva *et al.*, 2005 ▸; Garman, 2010 ▸; Owen *et al.*, 2012 ▸).

Despite these differences in damage mechanism between cryo-temperature and room temperature, the specific damage artefacts observed at both temperatures are broadly similar. Metal-ion and cofactor reduction, as well as disulfide reduction, have been observed in multiple RT radiation-damage studies (Helliwell, 1988 ▸; Southworth-Davies *et al.*, 2007 ▸; Ebrahim *et al.*, 2019 ▸; De la Mora *et al.*, 2020 ▸). Furthermore, the onset of these artefacts is typically observed at lower doses in RT compared with cryo-temperature data sets. The onset of cofactor damage has been observed at tens of kGy in RT data collection (Ebrahim *et al.*, 2019 ▸); this is similar to the doses at which cofactor damage has been detected at cryo-temperatures (Ueno *et al.*, 2019 ▸), but results from the fact that reducing the dose below a few kGy whilst still collecting a complete, high-resolution data set is currently impractical. However, at RT the onset of disulfide-bond damage has been observed at doses as low as 10 kGy (De la Mora *et al.*, 2020 ▸), although notably there have been considerable discrepancies between the doses at which disulfide damage has been reported in different studies, with one study of insulin detecting no disulfide damage at doses as high as 500 kGy at RT (Roedig *et al.*, 2016 ▸).

As yet, aspartate and glutamate decarboxylation has not been observed in RT data sets. It is hypothesized that this results from the fact that the onsets of global and specific damage are observed at more similar doses at RT, and so by the time that aspartate and glutamate residues begin to be decarboxylated the crystal lattice has degraded sufficiently such that diffraction data can no longer be collected. Likewise, side-chain disordering due to RD has also not been observed at RT. Consequently, RT data sets capture the conformational heterogeneity of protein side chains much better than cryo-temperature data sets, in which side-chain conformations are known to be affected by both the low temperature and RD (Fraser *et al.*, 2011 ▸; Russi *et al.*, 2017 ▸; Yabukarski *et al.*, 2022 ▸).

In addition to proteins, nucleic acids are also known to suffer X-ray-induced radiation damage. However, to date only a small number of studies have examined the specific RD suffered by nucleic acids during MX data collection. Consequently, if an equivalent hierarchy of specific damage artefacts exists for nucleic acids as for proteins, it has not yet been established. Nonetheless, there is consensus between studies that nucleic acids are less susceptible to RD than proteins at cryo-temperatures (Fig. 3 ▸ *c*): the onset of specific damage artefacts is observed at a higher dose for nucleic acids when comparing nucleic acid and protein crystals subjected to the same experimental conditions (Bugris *et al.*, 2019 ▸) and in crystals of protein–nucleic acid complexes (Bury *et al.*, 2015 ▸; Bury, McGeehan *et al.*, 2016 ▸). These studies, in combination with theoretical and experimental radiation chemistry studies of nucleic acids in solution, also suggest that the sugar-phosphate backbone is more susceptible to RD than the bases (Sanche, 2005 ▸; Simons, 2007 ▸; Ptasińska & Sanche, 2007 ▸; Bury *et al.*, 2015 ▸; Bury, McGeehan *et al.*, 2016 ▸; Bugris *et al.*, 2019 ▸). More research is required, however, to determine the specific RD artefacts suffered by nucleic acids and their relative order (if any) of susceptibility in cryo-temperature and especially in RT MX studies.

Specific RD artefacts can be difficult to distinguish from biologically relevant structural features. In particular, RD can make it very difficult to accurately determine the structure of a redox-sensitive cofactor, which is often highly important when deducing the mechanism of action of the protein or other macromolecule to which it is bound. Several recent studies have analysed the X-ray-induced changes to cofactors that occur, even when the crystal sample is cryocooled, at doses of just tens of kGy (Ueno *et al.*, 2019 ▸; Zárate-Romero *et al.*, 2019 ▸; Pfanzagl *et al.*, 2020 ▸; Tandrup *et al.*, 2022 ▸). Consequently, it is often informative to use complementary techniques, such as online UV–Vis or Raman spectroscopy, to track changes in the excitation state, redox state and/or structure of cofactors during X-ray diffraction data collection (Cohen *et al.*, 2016 ▸; Gotthard *et al.*, 2019 ▸). X-ray free-electron lasers (XFELs) are also becoming increasingly popular for the collection of damage-free diffraction data from macromolecular crystals that are particularly sensitive to RD (Hirata *et al.*, 2014 ▸; Halsted *et al.*, 2019 ▸; Rose *et al.*, 2021 ▸): notably, however, RD has been observed in XFEL structures collected using longer and/or more intense pulses (Nass *et al.*, 2015 ▸, 2020 ▸; Galli *et al.*, 2015 ▸; Dickerson *et al.*, 2020 ▸).

### 3.2. How to identify specific damage artefacts in your data

Given the aforementioned difficulties in distinguishing specific damage artefacts from structurally relevant features, it is important for crystallographers to be aware of the confounding effects that RD can have on MX structures. To help with this, several programs have been released to enable users to identify specific RD artefacts within their own data and those of others. In order to distinguish RD from other artefacts, these programs require as input a model that fits well overall to its corresponding electron-density map and hence to the diffraction data. Accordingly, these programs should only be run towards the end of refinement.

Programs written to detect conformational heterogeneity/mismodelling have proven to be useful in the detection of conformational disorder induced by radiation damage in 2*mF*_obs_ – *DF*_calc_ maps (Lang *et al.*, 2010 ▸; Yabukarski *et al.*, 2022 ▸) and using atomic *B*-factor values (Masmaliyeva *et al.*, 2020 ▸). However, the most common method of detecting specific RD artefacts is to measure differences between successive diffraction data sets collected from the same crystal(s). Owing to the amount of time required to collect data, it used to be the case that unless they were studying radiation damage, a crystallographer would not usually collect more degrees of data than were required for a complete data set. However, the greatly increased speed of data collection enabled by the new generation of pixel detectors has allowed the collection of 360° of data to become standard practice, meaning that most data sets now contain a minimum of twice the amount of data required for completeness.

To calculate a difference map, a crystallographer requires a model that has been well refined to a data set *A* of scaled and merged reflections, plus a second data set *B* of scaled and merged reflections (which may overlap with/be a subset of data set *A*). As an example, data set *A* could correspond to the complete 360° of data collected, whilst data set *B* could correspond to the first 180°. A difference map is then calculated by subtracting the electron-density map calculated for the model and data set *B* from the map calculated for the model and data set *A*. This difference map can be inspected for the specific damage artefacts illustrated in Fig. 3 ▸(*a*): disulfide-bond reduction, for instance, is characterized by negative difference density around the bond between the two sulfurs (and sometimes in higher resolution maps positive difference density can indicate where one or both of the cysteine side chains have moved following bond cleavage if they have adopted defined conformations), whilst aspartate and glutamate decarboxylation is characterized by negative difference density around the side-chain carboxyl group.

As its name suggests, a difference map enables a researcher to measure the difference in damage between two data sets. Consequently, in the example above, the difference map calculated by subtracting data set *B* from data set *A* allows the identification of damage accrued in the second 180° of data collection; however, it does not provide information about any damage that may have occurred during the first 180° of data collection. Nevertheless, since nowadays most crystallo­graphers use 360° of data for structure solution, a difference map calculated against the first *X*° of data (where the minimum angle for a complete data set ≤ *X* < 360°) is a useful tool to check that the 360° of data that they are using does not contain damaged images. If the difference map does show evidence of damaged residues, however, this only tells the crystallographer that the first *X*° of data are less damaged than the subsequent (360 − *X*)° of data; it does not indicate whether the first *X*° of data contain damage artefacts or not.

A range of software now exists to allow the calculation and inspection of difference maps. The *autoPROC* software (Vonrhein *et al.*, 2011 ▸), an automated pipeline for processing diffraction data from raw images into scaled and merged intensity values, has recently been updated to include calculation of *F*_obs(early)_ and *F*_obs(late)_ data sets (Vonrhein *et al.*, 2024 ▸). The *F*_obs(early)_ and *F*_obs(late)_ data sets comprise images collected at the beginning and end, respectively, of the entire data collection, with each data set incorporating the minimum number of images required for completeness. This approach maximizes the distance in image space (and thus in dose) between the two data sets and hence maximizes the ease of detection of any RD artefacts that are acquired during the entire data collection. The *BUSTER* model-refinement pipeline (Bricogne *et al.*, 2023 ▸) can subsequently combine these data sets with a refined model to generate an *F*_obs(early)_ − *F*_obs(late)_ difference map for the user to inspect. Note that the difference maps presented in RD studies are typically *F*_obs(late)_ − *F*_obs(early)_ maps, hence damage artefacts in *F*_obs(early)_ − *F*_obs(late)_ maps display the inverse difference density changes to those shown in Fig. 3 ▸(*a*): *i.e.* in an *F*_obs(early)_ − *F*_obs(late)_ map a damaged disulfide bond will show positive difference density around the sulfur–sulfur bond and negative density where the sulfurs have moved to, and so forth. *AutoPROC* and *BUSTER* are both available as part of the *CCP*4 software suite (Agirre *et al.*, 2023 ▸), as well as being available to download from the Global Phasing website (https://www.globalphasing.com).

**Figure 4:**
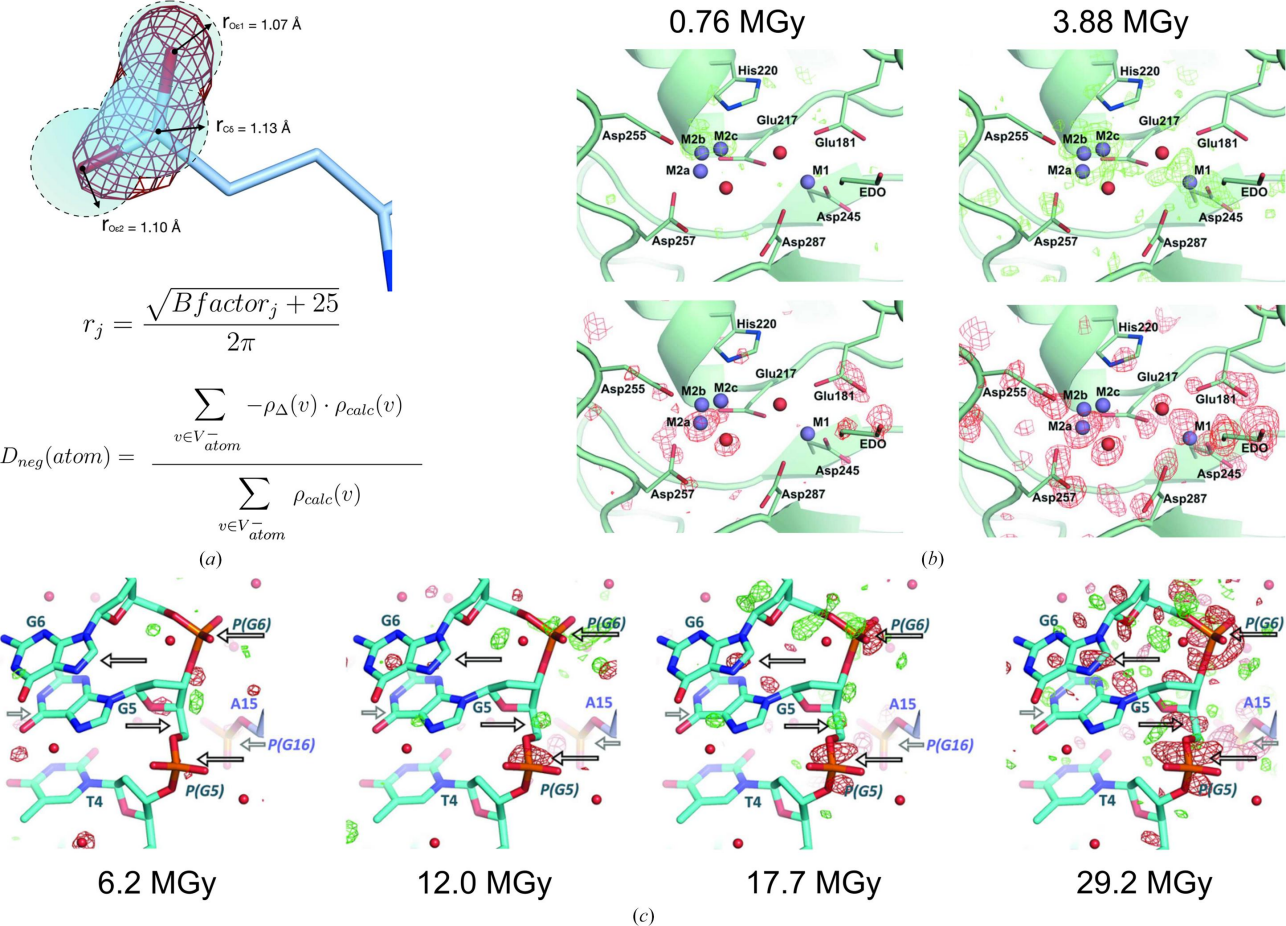
Identifying damage in *F*_obs(late)_ − *F*_obs(early)_ difference maps with *RIDL*. (*a*) *RIDL* calculates difference maps and divides them into voxels. The program measures the damage to atom *j* by first identifying the voxels within a radius *r_j_* of atom *j* and then calculating the *D*_neg_ metric (as well as several other metrics). ρ_Δ_(*v*) and ρ_calc_(*v*) are the density values at voxel *v* in an *F*_obs(late)_ − *F*_obs(early)_ map and a map calculated directly from *F*_calc_, respectively, whilst 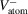 refers to all of the voxels with negative difference density in the *F*_obs(late)_ − *F*_obs(early)_ map within a radius *r_j_* of atom *j*. This figure was adapted from Bury *et al.* (2018 ▸). (*b*) Difference density maps produced by *RIDL* for a low-dose and a high-dose data set collected from a xylose isomerase crystal (PDB entry 6qrr; Taberman *et al.*, 2019 ▸); positive/negative difference density (contoured at ±3σ) is coloured green/red and shown separately in the top/bottom images for clarity. The xylose isomerase active site is shown, demonstrating the accrual of radiation damage in the higher relative to the lower dose data set. This figure was adapted from Taberman *et al.* (2019 ▸). (*c*) Difference density maps produced by *RIDL* for four data sets of increasing dose collected from a crystal of a DNA 16-mer (PDB entry 6qt1; Bugris *et al.*, 2019 ▸). Maps are contoured at ±3σ, with positive/negative density coloured green/red. Arrows indicate examples of sites where damage accumulates with increasing dose; damage artefacts are predominantly localized around the sugar-phosphate backbone. This figure was adapted from Bugris *et al.* (2019 ▸). All data leading to the structures presented in (*b*) and (*c*) were collected at 100 K.

A disadvantage of manual map inspection is that it introduces human bias into the detection and quantification of damage artefacts. It can also be intractable for very large proteins. To overcome these challenges, the *RIDL* software was written to systematically inspect difference density maps for RD artefacts (Bury & Garman, 2018 ▸). *RIDL* takes as input two scaled and merged data sets collected from the same crystal(s), plus a well refined model, from which it calculates a difference map. The program then divides the map into voxels and identifies the voxels within a sphere of radius *r* around each atom, with the value of *r* being determined from the *B*-factor value of the atom (Fig. 4 ▸ *a*). The *D*_neg_ metric for atom *j* is calculated as the weighted average of the negative difference density across its corresponding sphere of voxels (voxels with positive difference density are discarded from the calculation), with the negative difference density measured at each voxel being weighted by how much the density of atom *j* contributes to the total density measurement in an *F*_calc_ map (Fig. 4 ▸ *a*). Users can then rank atoms by their *D*_neg_ values to enable unbiased, rapid inspection of the difference map for RD artefacts. Fig. 4 ▸ shows examples of the *RIDL* output for xylose isomerase (Fig. 4 ▸ *b*; Taberman *et al.*, 2019 ▸) and a DNA 16-mer (Fig. 4 ▸ *c*; Bugris *et al.*, 2019 ▸), highlighting damage to the active site of the protein and to the sugar-phosphate backbone, respectively. *RIDL* is available on GitHub (https://github.com/GarmanGroup/RIDL).

There are several advantages in using difference maps to identify RD artefacts accrued within a data set. One of these is that difference maps can be calculated for data sets collected at any temperature; likewise, they can be calculated for a data set collected from any crystal species, irrespective of whether the crystal is a protein, a nucleic acid or another type of molecule. Accordingly, difference maps are the method of choice for researchers studying specific RD in MX.

There are, however, some drawbacks to difference maps. As described above, they can only be calculated for data sets that include more images than are required for completeness, and they are used to identify damage accrued between the *F*_obs(late)_ and *F*_obs(early)_ data sets; they do not provide information about the extent to which the *F*_obs(early)_ data set itself is damaged (if at all). Furthermore, difference map calculation requires access either to two different scaled and merged data sets or to the full unmerged data set: this is straightforward when a crystallographer is examining their own data, but is often not the case when examining the data of others. Since February 2008, crystallographers have been required to submit their scaled and merged reflection data alongside their refined MX model when depositing a crystal structure in the Protein Data Bank (PDB; wwPDB, 2007 ▸). However, they have the choice of whether they also submit their unmerged data (wwPDB Consortium, 2019 ▸). Most users just submit the merged data set, which precludes difference-map calculation. It is therefore not possible to run difference-map calculation software to assess all MX models in the PDB (Berman *et al.*, 2003 ▸) for damage artefacts.

**Figure 5:**
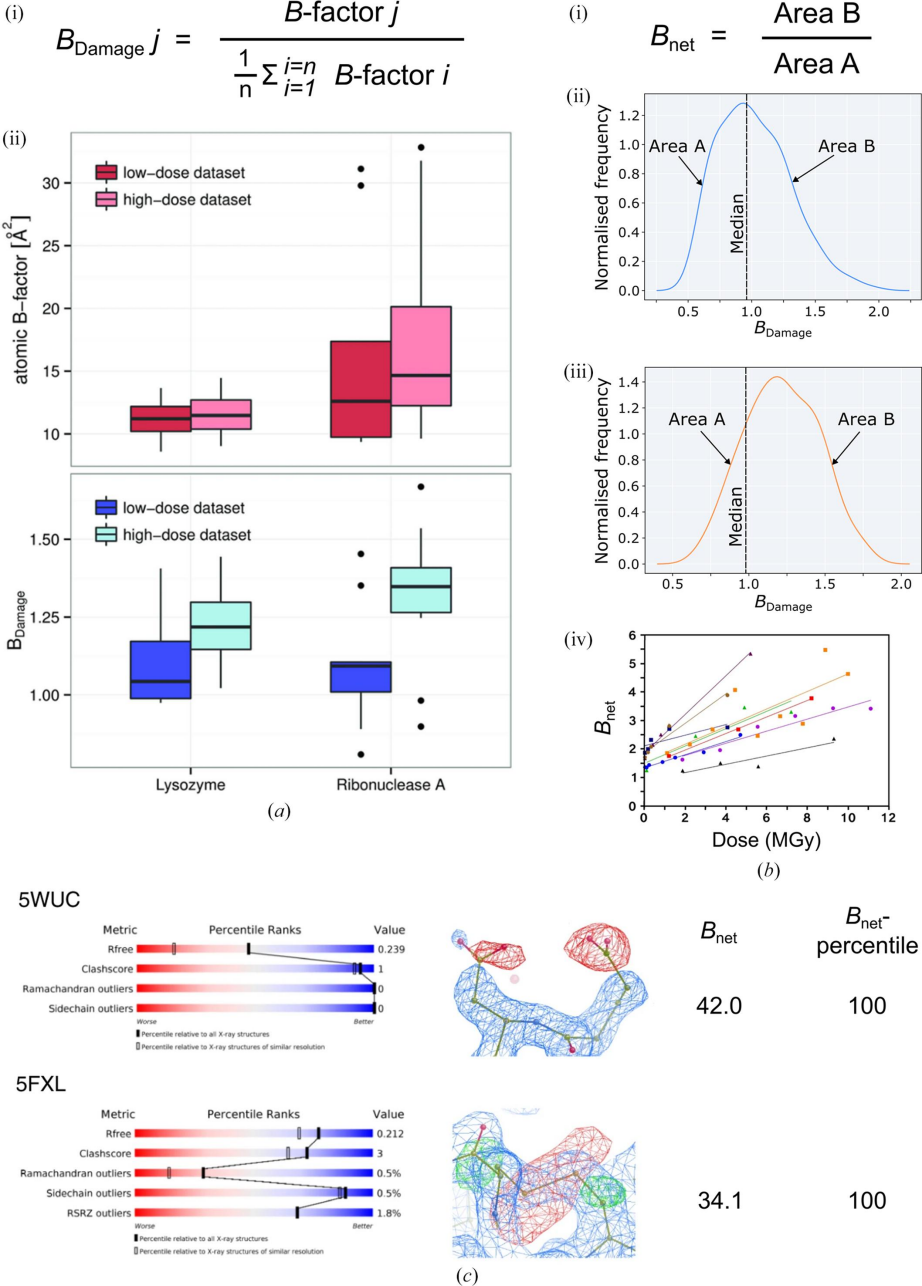
Identifying specific radiation damage with the *B*_Damage_, *B*_net_ and *B*_net_-percentile metrics in cryo-temperature protein crystal structures. (*a*) The *B*_Damage_ metric identifies atoms with high *B*-factor values relative to other atoms in a similar local packing-density environment in the crystal. (i) Definition of the *B*_Damage_ metric. (ii) Box plots of the isotropic atomic *B*-factor and *B*_Damage_ values for glutamate side-chain O atoms in low-dose and high-dose data sets collected from lysozyme and ribonuclease A (PDB entries 2blx, 2bly, 2blp and 2blz; Nanao *et al.*, 2005 ▸). Boxes represent the median and interquartile range (IQR), outliers are represented as black dots, and whiskers represent the range (excluding any outliers). This figure was adapted from Gerstel *et al.* (2015 ▸). (*b*) The *B*_net_ metric identifies structures whose aspartate and glutamate side-chain O atoms have high *B*_Damage_ values in comparison to the rest of the structure. *B*_net_ is calculated by plotting a kernel density estimate (KDE) plot of the *B*_Damage_ values of aspartate and glutamate side-chain O atoms. The area under the curve is calculated to the left (*A*) and right (*B*) of the median *B*_Damage_ value of all atoms in the structure; *B*_net_ is then calculated by dividing *B* by *A.* (i) Definition of the *B*_net_ metric; KDE plots demonstrating the *B*_net_ calculation are shown for (ii) a low-dose structure and (iii) a high-dose structure (PDB entries 5mcc and 5mcn, respectively; Bury *et al.*, 2017 ▸). (iv) *B*_net_ is correlated with dose. The plot shows *B*_net_ values calculated for nine radiation-damage series (PDB codes and references are provided in Shelley & Garman, 2022 ▸). These figures were adapted from Shelley & Garman (2022 ▸). (*c*) wwPDB validation statistics, models and density maps for two of the most damaged cryo-temperature protein crystal structures deposited in the PDB, as determined using the *B*_net_ and *B*_net_-percentile metrics. 2*mF*_obs_ − *DF*_calc_ maps (blue) are contoured at 1.5 r.m.s.d.; *F*_obs_ − *F*_calc_ difference density maps are contoured at ±3.0 r.m.s.d., with positive and negative density coloured green and red, respectively. This figure was adapted from Shelley & Garman (2022 ▸).

Whilst only a minority of structures are deposited with the necessary data for difference-map calculation, in contrast every structure is deposited with an atomic coordinate model. In this model, *B*-factor values record the uncertainty in the coordinates of each atom. Consequently, *B*-factor values contain information about RD artefacts: when an atom is damaged it moves, increasing the uncertainty in its position and hence also increasing its *B*-factor value. However, *B* factors are affected by numerous other variables, in particular conformational heterogeneity between the asymmetric unit copies in the crystal, which prevents *B*-factor values from being used as a direct readout of RD (Fig. 5 ▸ *a*).

The conformational heterogeneity of an atom is correlated with its packing density (the number of atoms in its local environment; Weiss, 2007 ▸). Gerstel and coworkers found that when they corrected *B*-factor values for packing density, the resulting ‘*B*_Damage_’ values of known sites of specific damage were correlated with dose (Fig. 5 ▸ *a*). *B*_Damage_ is calculated as the ratio of the isotropic *B*-factor value of an atom to the mean average isotropic *B* factor of the other atoms in the structure that are identified to be in a similar local packing-density environment (Gerstel *et al.*, 2015 ▸). Accordingly, the *B*_Damage_ metric identifies the atoms within a model that have the highest *B*-factor values in relation to their packing density. If a structure is damaged the atoms with highest *B*_Damage_ values will be sites known to be susceptible to damage, such as disulfide-bond sulfurs and glutamate/aspartate side-chain carboxyl groups. Conversely, if it is undamaged these sites will not be overrepresented amongst the atoms with the highest *B*_Damage_ values. *B*_Damage_ values can be calculated for MX structures collected at any temperature. Note, however, that because it is a *B*-factor-derived metric, *B*_Damage_ values should not be calculated for components whose occupancy (across all modelled conformers) could be less than one, such as small-molecule ligands.

A drawback of *B*_Damage_ values is that owing to the variability in the relationship between *B* factor and dose, *B*_Damage_ values cannot be fairly compared between different MX structures. The *B*_net_ metric was therefore developed to enable this comparison (Shelley & Garman, 2022 ▸). To achieve this, the *B*_net_ calculation plots the *B*_Damage_ values of the aspartate and glutamate side-chain O atoms of a protein alongside the median *B*_Damage_ value of all atoms in the model: the larger the values of the former relative to the latter, the greater the damage suffered by the structure (Fig. 5 ▸ *b*, i–iii). Since *B*_net_ uses damage to aspartate and glutamate side chains to summarize the extent of damage suffered by a model, it is consequently only suitable for assessing damage to protein structures collected at cryo-temperatures. In addition, for reliable results the analysis should only be carried out for models that contain more than ten glutamate/aspartate residues in the asymmetric unit.

*B*_net_ has been validated to be correlated with dose (Fig. 5 ▸ *b*, iv) plus found to be independent of the majority of variables other than dose that can affect *B*-factor values. However, *B*_net_ was found to be correlated with resolution and hence the *B*_net_-percentile metric was defined: *B*_net_-percentile is calculated as the percentile ranking of the *B*_net_ value of a model within the subset of models derived from cryo-data sets which are closest in resolution (the 1000 structures closest in resolution in the PDB are first identified, and subsequently all structures falling within this resolution range are included in the percentile calculation). *B*_net_-percentile is not correlated with resolution, and hence is the recommended metric for damage comparison between structures.

The *B*_net_ and *B*_net_-percentile metrics were used to analyse damage to 93 978 cryo-temperature protein crystal structures deposited in the PDB (Berman *et al.*, 2003 ▸) and PDB-REDO (van Beusekom *et al.*, 2018 ▸) repositories as of 19th November 2020. This study identified numerous damaged structures (Fig. 5 ▸ *c*), including several that are so damaged that the artefacts that are typically observed in difference maps were detectable in their individual *F*_obs_ − *F*_calc_ maps (damage artefacts are usually obscured in these maps due to additional noise; Shelley & Garman, 2022 ▸).

From this analysis, a *B*_net_-percentile value of 95 was found to correspond to a *B*_net_ value of approximately 3.0. These values are suggested as general thresholds above which a cryo-temperature protein crystal structure should be inspected carefully for RD artefacts: structures with *B*_net_-percentile values above 95 are amongst the top 5% most damaged cryo-structures in the PDB. However, the *B*_net_ and *B*_net_-percentile values that are acceptable to a user will depend on the application for which they are intending to use a structure. As an example, an analysis of dose versus *B*_net_ for 15 RD data-set series has revealed that a dose of 3 MGy, the dose around which the onset of aspartate/glutamate side-chain damage is observed, corresponds to *B*_net_-percentile and *B*_net_ values of approximately 85 and 2.5, respectively. A crystallographer wanting to draw conclusions about the apparent conformational heterogeneity of a catalytic aspartate residue might therefore wish to study structures with metric values below these thresholds. Researchers studying a redox-sensitive cofactor would need to select even lower thresholds: in this case it would be advisable to use complementary methods such as online UV–Vis or Raman spectroscopy to check for damage to the cofactor during data collection.

The *B*_Damage_, *B*_net_ and *B*_net_-percentile metrics can be calculated using the *RABDAM* software (Shelley *et al.*, 2018 ▸). *RABDAM* is available on GitHub (https://github.com/GarmanGroup/RABDAM) and is distributed as part of *CCP*4 (Agirre *et al.*, 2023 ▸). *B*_net_ values will also soon be reported in the PDB-REDO database (van Beusekom *et al.*, 2018 ▸).

If a crystallographer detects specific damage in their structure, they can often remove these artefacts by excluding images collected at the end of the data set – whilst maintaining sufficient completeness for structure solution – and then re-refining their model to this truncated data set. Damaged images can be identified using the metrics described in Section 2.2; however, since these metrics detect global damage, sometimes it may also be necessary to discard apparently undamaged images in order to remove specific damage artefacts. In many cases, this truncation will substantially improve the quality of the refined maps. Occasionally, though, it will not prove possible to remove all damaged images whilst maintaining sufficient completeness, in which case it would be advisable to collect a new data set.

## 4. A practical example

**Figure 6:**
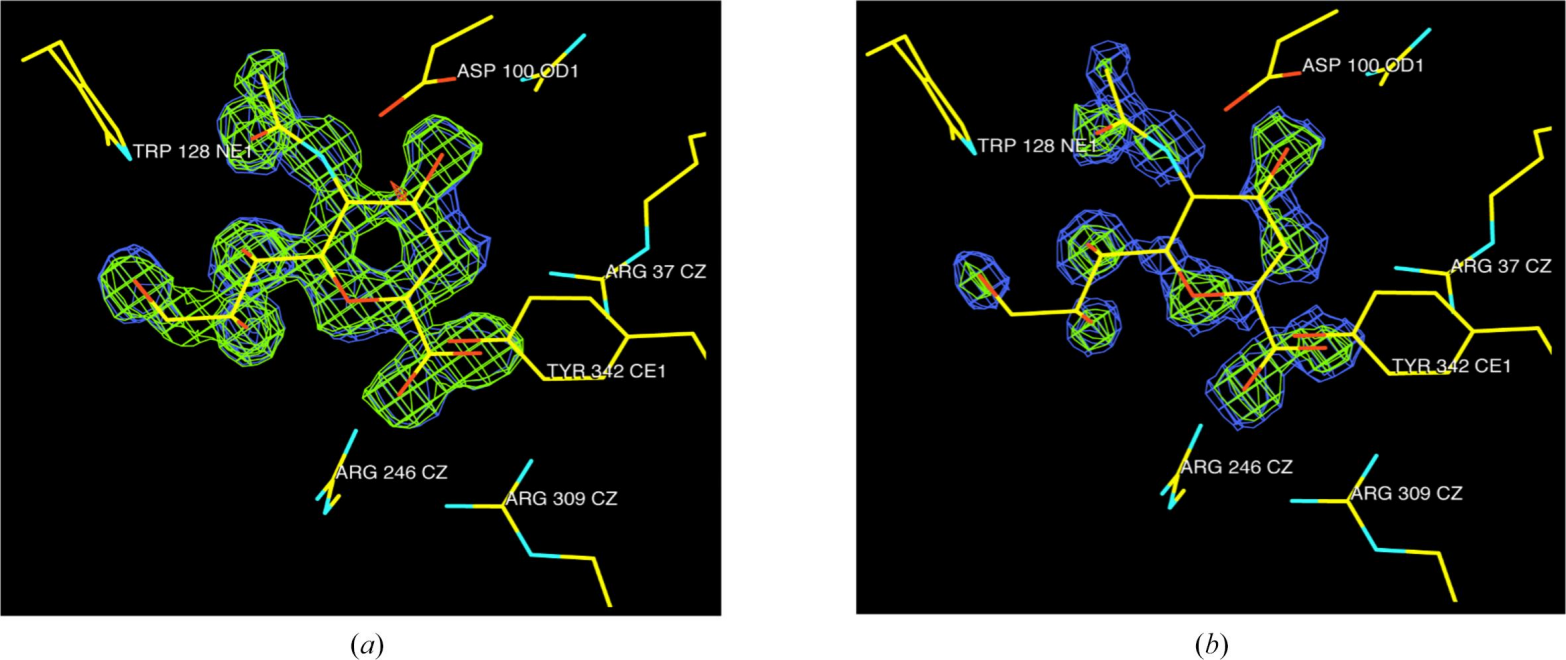
The ‘polluting’ impact that radiation damage can have on the electron-density map. 0.89 Å resolution data collected from an STNA sialidase D62G mutant crystal soaked in the transition-state analogue DANA. (*a*) 555 images and (*b*) 588 images including 33 that were clearly radiation-damaged (see the text).

An example of the ‘polluting’ impact that radiation damage can have on the electron-density map of an ultrahigh-resolution (0.89 Å) structure, resulting from the deleterious effects of damaged images on the scaling of the reflections, is shown in Fig. 6 ▸. The data were collected in the late 1990s at Stanford Synchrotron Radiation Lightsource (SSRL) on a circular MAR345 imaging-plate detector from a cryocooled crystal (110 K) of an active-site (D62G) mutant of sialidase from *Salmonella typhimurium* (STNA; PDB entry 7af2) which had been soaked in a transition-state analogue, DANA (2-deoxy-2,3-dehydro-*N*-acetylneuraminic acid). The strategy was to collect a ‘low’-resolution (2.66 Å at the detector edge, detector-to-crystal distance *d* of 551 mm) set of 160 images with an oscillation angle (Δφ) of 1.5°, a ‘middle’-resolution (8–1.35 Å, *d* = 250 mm) set of 129 images with Δφ = 0.8°, a ‘high’-resolution set (2.0–0.83 Å, *d* = 110 mm) of 182 images with Δφ = 0.6° and finally a ‘high2’ (2.2–0.86 Å, *d* = 120 mm) set of 116 images with Δφ = 0.5°. This gave 588 images altogether, which were scaled together using both *SCALA* in *MOSFLM* (Evans, 2006 ▸; Battye *et al.*, 2011 ▸) and *SCALEPACK* in *DENZO* (Otwinowski & Minor, 1997 ▸). The resulting electron density for *DANA* showed breaks (Fig. 6 ▸ *a*), but when the final 33 images were excluded *DANA* showed continuous high-resolution density (Fig. 6 ▸ *b*). Analysis of the scaling statistics showed that these 33 images were damaged enough to significantly compromise the data quality by affecting the way that the entire data set was being scaled.

## 5. Conclusions

It is clear that radiation damage has become more of a mainstream concern, about which all macromolecular crystallographers should be aware. With the increasing numbers of very high flux density beams becoming available at fourth-generation synchrotrons, RD to the samples will be even more prevalent. To circumvent this issue, it is anticipated that RT serial crystallography will become more widely utilized at these synchrotrons.

Given the deleterious effects that can manifest in the electron density of structures, the best approach is for the experimenter to avoid inflicting radiation damage on their crystals in the first place during data collection. Tools to aid this are increasingly becoming available at synchrotron beamlines, in terms of easy online dose estimators and fast auto-processing during the experiment.

However, if a data set does show damage artefacts once the structure has been refined (for example disulfide bonds, glutamates and aspartates appearing to lack electron density), all is not lost, since the damaged data can be removed by examining the statistics in reciprocal space and taking out the images where the processing statistics clearly show that the data are compromised. Additionally, if the *B*_net_-percentile value of the refined structure is greater than 95, the researcher should check their data set carefully for damage artefacts: they may wish to select a more stringent *B*_net_-percentile threshold depending on the biological interpretations that they are hoping to draw from the structure.

We hope that the increasing availability of tools to detect radiation damage will continue to increase general awareness of the problem and help users to identify and avoid it in their structures and those of others. However, there remains a pressing need for further research to develop high-throughput tools to identify RD at RT and also to detect RD in nonprotein components.
